# Caring for family members following suicide: Professionals’ experiences of responsibility

**DOI:** 10.1177/09697330221136631

**Published:** 2023-01-03

**Authors:** May Elise Vatne, Dagfinn Nåden, Vibeke Lohne

**Affiliations:** Department of Nursing and Health Promotion, 60499 Oslo Metropolitan University, Oslo, Norway

**Keywords:** Suicide, psychiatric ward, family members, professionals' experiences, ethics

## Abstract

**Background:**

When a patient commits suicide while hospitalized in the psychiatric ward, the mental healthcare professionals (MHCPs) who have had the patient in their care encounter the family members immediately following the suicide. Professionals who encounter the bereaved in this first critical phase may have a significant impact on the grieving process. By providing ethically responsible and professionally competent care, they have the opportunity to influence what can alleviate and reduce suffering and promote health in a longer perspective.

**Aim:**

The aim of this study is to investigate MHCPs’ experiences in the encounter with family members who has been bereaved by suicide.

**Methods:**

Data material consists of text from in-depth interviews with six MHCPs belonging to a total of five different psychiatric units in two hospitals. The findings have emerged through analysis using a hermeneutical approach based on Gadamer’s philosophical hermeneutics.

**Ethical considerations:**

The study was approved by the Ombudsman for Privacy of the Norwegian Social Science Data Services and is based on informed consent and confidentiality.

**Findings:**

Three themes emerged: Confirming the suffering. Creating encounter through dialogue. Providing consolation and reconciliation. Findings illuminate how MHCPs understand their responsibilities and how they act in the encounter with the bereaved following suicide.

**Conclusion:**

The participants appear to be led by the responsibility that grows through witnessing the suffering of the bereaved. Encountering the family member’s aggression and threats against staff members is an ethical challenge to the professional’s ability to confirm the bereaved, create dialogue and provide consolation and reconciliation at the start of their grieving process. MHCPs need to be aware of the different reactions and needs of family members following suicide. More research is needed about how to provide sensitive and flexible care in ways that can be perceived as helpful for those left behind.

## Introduction

In Norway, 639 suicides were recorded in 2020, a number in line with the rest of Europe, North America and Australia.^1^ Suicide also occurs in conjunction with hospitalization in a psychiatric ward. The risk peaks in periods immediately after admission and discharge.^2^ At some point, most professionals working in the mental health field will experience patients committing suicide during the course of treatment.^3,4^ Health and mental health professionals may experience reactions such as shock, guilt, sadness, anxiety and shame. They may also begin to doubt their professional competence.^4^ In line with police, clergy, ambulance personnel and others, they will meet family members immediately after a suicide and may have a significant impact on the subsequent process. Response to the bereaved will be positively affected by the professional’s experience and security related to suicide.^5^

Mental healthcare professionals’ (MHCPs’) encounter the family members when a patient previously in their care has committed suicide. This study contains these professionals’ experiences caring for the bereaved family members.

### Background

Each year, between 5000 and 6000 family members or other close associates are affected by suicide.^1^ The Norwegian Directorate of Health^6,7^ provides recommendations and advice on follow-up of the bereaved by suicide. Suicide ends one person’s unbearable suffering but brings new suffering to those left behind.^8,9^ Research shows a wide range of reactions in the bereaved by suicide. Shock, sorrow, pain, missing and longing to be reunited with the deceased are reactions in line with those of other bereaved after unexpected death, according Andriessen et al.^9^ Distinctive reactions are anger at the deceased, aggression and feelings of abandonment and rejection. The recurrent themes in narratives of bereaved after suicide include guilt, shame, social stigma, a search for meaning, and suicidal ideation.^9^

Providing help to humans bereaved by suicide is a direct form of suicide prevention.^5,9^ The process after losing a loved one to suicide is painful and just as unique as a ‘fingerprint’, and for some it also poses a risk of suicide.^10^ Mothers who have lost a son or daughter to suicide are particularly vulnerable to dying in the same way.^11^ Parents who experience a son’s or daughter’s suicide attempt describe this experience as ‘a double trauma’,^12^ because the traumatic event also has psychosocial consequences for the parents’ health and life. The grieving process is individual in nature, and the focus is often individuals grieving alone, although they rarely grieve totally isolated from others.^13^ Family dynamics could be affected, as the person grieving oscillates between the experience of loss and mastering the change in one’s role, identity and relationship on their way to new orientation.^13^

Several studies show that the need for support for family members following suicide is varied and changes. Many people need professional and often long-term follow-up to go on with their own lives.^14–16^ Adolescents who have been followed up by professionals emphasize the time aspect: They may need more help in the long term rather than immediate help after the suicide.^16^ Family members report the importance of not feeling alone, especially during the critical period after the suicide. They need support in a social network and in the company of peers,^11,14,17^ they need to encounter their own barriers against seeking assistance, and they need professionals to initiate support and appropriate help measures.^11,18^ When people who have lost a family member to suicide seek help from a professional who knew the patient, the professional’s attention to reactions and signs of complicated grief may overshadow their alertness to potential suicide risk among family members.^17,19^

Relational factors in contact with professionals appear to be of great importance in the care of family members following suicide. Studies show that the ability of professionals to show understanding and compassion leave positive traces.^18^ Adolescents must perceive that they are understood and appreciated, and that they are able to decide on their own when they are ready for professional help.^15,16^ Compassion, embracing, listening, sensitivity and flexibility are regarded as important components in caring for those left behind.^20^

To our knowledge research on experiences with the bereaved family members following suicide is scarce within the field.

### Aim of this study

The aim of this study is to investigate MHCPs’ experiences in the encounter with family members of patients following suicide.

## Theoretical framework

This hermeneutical study is based on Gadamer’s philosophical hermeneutics^21^ and Eriksson’s caring theories.^22–24^ Hermeneutics is characterized by sensitivity and receptiveness to what reveals itself in the process of discovery, which in turn can engender new questions so that existing understanding is challenged and expanded.^21,25^ According to Gadamer,^21^ to understand a matter requires openness to the other person’s or the text’s perceptions, but also being able to relate the other’s perceptions to the entirety of one’s own perceptions. Understanding is about understanding the matter differently, by reading texts from the part to the whole and back to the part.^21^

Suffering is a basic concept in caring theories, according to Eriksson.^22–24^ Suffering is part of being human and affects existence and meaning and is unique for each individual.^22–24^ The experience of not being seen by others, of being considered ‘dead’ is perhaps the deepest form of suffering a person can experience, because such experiences do produce an intense feeling of loneliness.^22,23^ The caring in listening, confirmation, consolation and strengthening hope is vital for human beings’ health and for alleviating suffering,^22–24,26–28^ not least in family members bereaved by suicide, who are left with deep despair and often guilt. Encountering is an act of care that can release inherent forces and influence movement towards health, according Lindstrøm.^26^ An adequate and compassionate encounter with painful experiences and feelings requires a sensitivity to the patient or family members’ suffering and requires ethical responsibility and courage to give something of oneself.^22,23^ Giving the suffering space confirms dignity and is crucial for being able to relieve the human being’s deep suffering.^22,23,28–30^

Based on this, the research question is: How do MHCPs in psychiatric wards describe and understand their responsibilities and tasks in their encounter with family members following patients’ suicide

## Methods

### Recruitment, context and participants

This study is a part of a larger project with focus on MHCPs’ experiences caring for family members when a patient at risk of suicide is hospitalized in a psychiatric ward.^31^ A request to interview health personnel was sent to the senior clinical leader in two hospitals having acute psychiatric functions. The inclusion criteria for participation in the study were: Both men and women, nurses and others with a 3-year health and social work background and with specialized studies in mental health care, a minimum of 5 years’ experience in mental health care, and experience in having contact with family members of patients at risk of suicide. The leader made sure that everyone who met the criteria for participation received information about the project and could register their interest. One of the researchers received the names of interested parties either from employees themselves or from one of the staff members who had a role as coordinator.

Six MHCPs, three men and three women, were included in this study. The participants comprised one social worker and five nurses. In addition to specialized studies in mental health care, some of these had further education in other fields such as family therapy and group therapy. The average sum of work experience in mental health was 14 years, primarily in the specialist health service and acute psychiatric wards. Most participants had lengthy experience in the hospital where they were employed. They all had follow-up families and relatives following suicide. The participants belonged to a total of five different units. Apart from two of them who possessed different special functions, they all worked as milieu therapists.

### Data collection

The data material consists of texts from individual research interviews conducted at the participants’ workplace, audio-file recorded and transcribed by a company certified to do this. The interviews lasted between 60 to 90 minutes each. They were prepared and carried out in accordance with Kvale’s conception of the interview as a research conversation^32^ and were conducted based on an interview guide. According to Kvale,^32^ a guide can help to make the conversation planned and flexible in order to facilitate an open and dynamic dialogue and thereby a rich body of material.

The opening question invited the participants to relate a situation in which they had an encounter with one or several family members of a patient who had committed suicide. Other questions concerned how they thought about caring for the bereaved family members, and what they experienced as challenges and dilemmas in caring for family members following suicide.

### Data analysis

The researchers individually read all interviews to familiarize themselves with the texts and with what the participants intended to convey. In the quest for themes that could illuminate the research question, the individual interviews were then read and reread by the researchers together as a group in order to share ideas about potential themes. At the same time, text passages were marked when they were deemed appropriate to document various themes. The content, understood as responses to the questions in the interview guide, was, in an early phase in the analysis process, presented in the resource group affiliated with the project. This group is explained in the section Strengths and limitations.

The data material concerns the participants’ experiences encountering one or several of the family members after a patient had committed suicide while he or she was admitted to a psychiatric ward. Some interviews concern experiences from several suicides; one of these occurred at a participant’s previous workplace. The six interviews, amounting to a total of about 180 A4 pages, constituted a rich data material and we assessed this as fully valid to illuminate the research question. The interviews were further analysed in a search for potential themes through dialogue with the text. During the work to present results, the themes became more distinct and were assigned a name. In this phase, we went back to the data to check whether the quotations documented the name. Table 1 illustrates the process of searching for potential themes leading up to the three themes which are this study’s findings. A hermeneutical approach to the interview texts, according Gadamner,^21^ implies a receptivity to a different understanding, until the analysis is understood as consistent, and the selected quotations seem to give meaning to and substantiate the themes.

**Table 1.** Phases in the analysis process.

Examples of quotations	Searching for potential themes	Themes
*You see how despairing the family members are*	Encountering the family members’ suffering and their various expressions of feelings and shock reactions following suicide, by	Confirming the suffering
*You see the family members ‘disintegrating’*		
*‘How could this happen?’*	Compassion and listening to their despair	
…*Accusations were made such as, ‘you took her life’, ‘I will never forget this’, ‘major consequences will come of this’, ‘I hope you won’t be able to sleep at night’; in other words, those kinds of statements.*	Embracing anger and accusations against the staff	
*They were shown the room where it had happened. We explained actually in detail what had happened, and they were also very preoccupied with how it could happen on a ward, and we explained how we worked on the post, and … I experienced that during that process the encounter became better and better. I think they experienced that we were genuinely concerned about how they were feeling after this had happened.*	Using procedures as a starting point for dialogue, by	Creating encounter through dialogue
	Talking about what had happened	
	Providing information about the therapy: What had been thought and done, and why	
	Providing time and space for questions	
*We never got in a position to start a very good dialogue. There were a lot of attacks on us, in a way*…	Listening to and embracing reactions	
	Initiating dialogue even in a locked situation	
*I think it is incredibly important for processing to be able to grieve and to get consolation, because there is a lot of emotion present, a lot of sorrow and a lot of pain*…	Follow-up in the first phase after the suicide, by	Providing consolation and reconciliation
	Having encounters with those who knew the patient, if the family members so wish	
*We talked a lot about this patient's personality traits, core characteristics, and what I greatly admired about him. I think that is good for them* …	Initiating contact and repeating invitations	
*I think it may be a little difficult for us to realize how long this grieving can take*	Contributing help and support in the continuing process	

### Ethical considerations

The participants were especially cautious about not identifying patients, family and staff members by name and personal details, because inpatient suicide is a rare event. A total observance of the duty of confidentiality will always be a challenge in research involving questions about personal experiences. Information that might help identify persons was therefore either omitted or anonymized, even at the risk of losing some of the meaningful content. In research interviews, some moral responsibility is also a given necessity.^33^ We invited the participants to share personal experiences and reflections in openness and trust that requires the researcher to be sensitive to the participant’s integrity and dignity.

The study was approved by the Ombudsman for Privacy of the Norwegian Social Science Data Services. The participants gave written consent, based on written and oral information about the project, including anonymization and the right to withdraw from the study.

## Findings

In this study, three themes emerged from the text: (1) Confirming the suffering, (2) Creating encounter through dialogue, (3) Providing consolation and reconciliation. The themes illuminate how MHCPs understand their responsibilities and how they act in the encounter with family members bereaved by suicide. The themes are presented and documented by summarized texts and selected quotations.

## Confirming the suffering

This theme is about encountering family members’ shock reactions and feelings in the first phase after a suicide. The participants attempted to confirm suffering by listening to, embracing and tolerating what is expressed. The suicides occurred either during leave from a psychiatric department, just after discharge, or while on the ward itself. In cases where the suicide occurred on the ward, the drama of crisis management, notifications and coordination of measures were also described, as well as care for the welfare of colleagues.

Participants witnessed family members disintegrating emotionally; quiet weeping, outbursts of despair, speechlessness, anger, accusations and threats against staff members. One participant described a first encounter with a family:

*First, they were in shock and completely shattered and in despair. And then they were also quite angry. That is, one parent was angry, the other was more emotionally hurt. Or, they had different ways of expressing it. And then the siblings also reacted in different ways. (Participant no.5)*

*We didn’t see that coming*, the participants said about suicide in which they had the responsibility to follow up the family members. *It is actually not possible to predict suicide,* one of the participants said. *Suicide risk is assessed for all patients,* … *but all in our wards have risk factors. Fortunately, few of them take their own lives.* In some suicides the patients might have talked about not wanting to live, but then there were many other things in their accounts that spoke in favour of their wanting to live, the participant said. *Then there are others*, *who don’t take their own lives. These,* the participant said, *vent much of the pain that they can’t bear to keep to themselves. And we worry about them, from time to time*. Suicide and the encounters with family members who were left behind affect the participants: *It’s tragic when that is the result you get. This is what we are constantly working to avoid. It’s really tough; we’re just people working here*, as one participant put it.

The participants primarily described meetings with a lot of aggressive communication and in which there was a lack of dialogue with family members, or the encounter was complicated. *It was a terrible conversation. We were completely ‘exhausted’ afterwards*, said one of the participants about such a meeting. Another participant talked about a conversation with a similar starting point: Intense anger and accusations, and occasional attacks. The family had been very concerned and worried about the patient without explicitly mentioning suicide and were critical as to whether the staff followed up the patient closely enough. Therefore, they went into the meeting somewhat forewarned,

*but I was in no way prepared for how intense and how… I would almost use the word ugly… that the attack was; that is, personally directed at my colleague. …Accusations were made such as, ‘you took her life’, ‘I will never forget this’, ‘major consequences will come of this’, ‘I hope you won’t be able to sleep at night’; in other words, those kinds of statements. (Participant no.6).*

The participant described the emotional statements as ‘attacks’ that continued in the next meeting as well. They were unable to have a good dialogue with this family, and they were soon scheduled to be followed up by others. One of the family members later asked for a conversation, and the participant experienced that they had a good dialogue. Several of the participants relate situations having about the same starting point: intense anger and accusations against the staff for misevaluating the patient and the situation, and threatening statements like *‘you probably know that this is going to have huge consequences*’, but where the outcome was different after the process was given some time.

## Creating encounter through dialogue

The participants mentioned conversations with parents, spouses, siblings, children, grandparents and other relatives after a suicide, during which they tried to establish a climate for dialogue. They seemed to use points in national guidelines and local procedures as the point of departure for this type of conversation. One participant told how they worked through five or six meetings with a family after the suicide, which started initially with intense anger toward the staff:

*They were shown the room where it had happened. We explained actually in detail what had happened, and they were also very preoccupied with how it could happen on a ward, and we explained how we worked on the post, and … I experienced that during that process the conversation became better and better. I think they experienced that we were genuinely concerned about how they were feeling after this had happened. We talked a lot about how it could happen, what our thoughts were about it. We also stated quite clearly that just like when a patient is admitted with cancer or a heart attack, mental health care likewise cannot guarantee a cure for everything. (Participant no.6)*

The above quote reflects repeated meetings that contained factual information and gradually encounters where two parties talked together about the suicide from the perspective of both parties. Another participant, however, reported conversations in which dialogue was never established:

*We never got in a position to start a very good dialogue. There were a lot of attacks on us, in a way. We are prepared for that; after all, it does happen occasionally, but … We had a conversation with other members of the family afterwards. And they also had a lot of questions, but this time we were allowed to tell our story and how we experienced the incident, what treatment we gave, without constant interruptions and without being contradicted about everything that we said. We established a dialogue; we were able to talk about the patient and about the treatment. We were able to say what we wanted to say, and they could express what was on their mind. (Participant no.3)*

When they experienced being unable to achieve the dialogue they strive to establish, it felt to them as if they had lost the opportunity to give family members something they thought could help them later.

*No one has learned how to deal with such crises.* The statement comes from a participant who claims, based on crisis theory, that it is unlikely, after a suicide, that bereaved will be able to absorb everything they are informed about:

*They don’t know exactly what they need to talk about, either. What about ensuring that they know about LEVE (The Norwegian Organization for the Suicide Bereaved)? That’s not exactly one of the things you say in the first conversation. What about children, care support groups? What about family counselling services? And in an initial conversation, you don’t have a chance to convey this. (Participant no.2)*

To inform family members in shock about various emergency services in a first meeting was not perceived by the participants as either possible or professionally responsible. They sought first to confirm and embrace their suffering and to build confidence as a basis for dialogue.

## Providing consolation and reconciliation

The participants talked about encountering family members following the patient suicide as experiences that had affected them emotionally. They described follow-up of the bereaved by suicide as a task they prioritized. They expressed a wish to provide something, either emotional or practical, that could help the bereaved to progress in their grieving process. The participants mentioned examples of support such as help to contact a close friend in the crisis, to submit their case in a written complaint to the County Governor, or to ensure that bereaved children in a family were followed up. Consolation was one of the care components that was highlighted:

*I think it is incredibly important for processing to be able to grieve and to get consolation, because there is a lot of emotion present, a lot of sorrow and a lot of pain. And then I think that consolation may be at any rate the right thing at the right time. (Participant no.5)*

Most family members wanted to talk to someone who had known the patient, the participants said. In some cases, the participant had had a longer relationship to the patient who had committed suicide. This allowed them to tell how they perceived the patient and assessed the situation. The participants described examples of messages in which they had shared their personal opinions about the deceased. *He was a wonderful person, a really nice guy*, one participant said about a patient who had committed suicide. *We talked a lot about this patient’s personality traits, core characteristics, and what I greatly admired about him. I think that is good for them*, the participant added.

In some cases, follow-up was described as lasting over two years, or extending over three years with less frequent meetings over time. *I think it may be a little difficult for us to realize how long this grieving can take*, one of the participants said. They gave examples of how grieving moves in waves and that many bereaved by suicide take advantage of the offer to be in contact. So they were keen to convey that the door was open, as in this case:

*… I think the pain this parent is left with is quite heavy. That’s why they won’t let go of me but want us to meet from time to time. And so I just say, ‘OK, let’s do that’. (Participant no.2)*

Several of the participants had examples of long-term work for those who are bereaved by suicide. They had experiences with family members who struggled with questions for a long time after the suicide. Follow-up at the hospital where it happened provides opportunities for follow-up that they otherwise don’t have, one participant said, as in this case:

*So I’ve asked the doctor from here to join our conversation, because a grandmother needed to talk more about what happened and about the treatment. And of course, you can do much better when they have contact with people here where the treatment took place. So if you think of the case as the patient who died, and then having several children, the public health nurses, family counselling, with each and every one, with several other people, with children, with the department head, with the doctor involved in the treatment; all of this is not written down anywhere. (Participant no.2)*

This participant reported that they made brief entries in the deceased patient’s chart*, but it does not appear in any procedures or codes or interventions, or maybe not at all.*

Participants emphasized the importance of professionals initiating contact with family members and repeating the invitation:

*If they say, ‘No, we have so many; we have good support’ and so on. Then I think we should say, ‘OK, listen, we’ll call you after a month and find out how things are going with you.’ That’s what we should do. We shouldn’t say, ‘You can just call us’. (Participant no.2)*

The participants mentioned the use of SMS and E-mail to remind families that they are still welcome to come in for conversations, even though the use of those kinds of communication media goes beyond the department guidelines. When dialogue in the critical phase following a suicide fails, it is considered particularly important to lower the threshold for contact.

*When is enough follow-up?* is the question asked by one participant, who mentioned a spouse who reported that he was now receiving good follow-up and that the children were well taken care of: *He states clearly that from now on I will no longer need to contact him. But I can imagine that the threshold for getting in contact is high.* The participant therefore considered contacting the spouses in a few months’ time. We always end contact with the bereaved after a suicide by saying *that we are here, and it is possible to contact us again.* They have experienced that even in cases where staff members have made every effort, relatives may still later report that they did not receive sufficient follow-up. *You have to stop at some point, but it can be difficult to know where the limit goes for what the relatives may experience as ‘pestering’*, in the words of this participant.

## Discussion

Findings in this study show that the professional’s encounter with family members in an early phase after the suicide in a psychiatric unit can be a demanding caring task. The theme *Confirming the suffering is* understood as an important caring act, where MHCPs’ encounter with the families and other relatives included strong emotions. In line with Norton,^5^ the first time after the suicide offers a critical opportunity for reducing the risk of suicide in the bereaved and for promoting health. The participants’ ethical responsibility seemed to be awakened by facing this suffering, according to Eriksson.^22,23^ They sought to confirm the strong expressions. Confirmation is, as we see it, that professionals take the perspective of the family members by listening to what is expressed and the implied meaning behind it, and also embracing and tolerating strong emotional outbursts. Confirming means validating the other, Lindström writes; validation is precisely the foundation of confirmation.^26^ Encounters characterized by listening and confirmation may release a force that helps recognize suffering.^26,28,30^ Grad and Andriessen^10^ consider recognition of feelings related to suicide as an important part of the process of moving on in their own lives. In our view, the experience that someone else embraces strong feelings can help family members to embrace their own feelings, in the long term. Embracing is considered the most important task in the encounter with relatives of patients at risk of suicide.^31^ This task has at least equal relevance when the suicide that is sought to be avoided has actually occurred.

The participants are regarded as working in accordance with Eriksson’s caritative caring theory, where confirmation is motivated by sensitivity and compassion.^22–24^ To confirm is to touch the other, Lindström claims,^26^ and poses the question as to whether it is possible to confirm another person without being affected oneself. Bereaved family members report having experienced a lack of respect and sensitivity in meetings with professionals, but also compassion and attentiveness.^18^ Quotations show that even though the participants are prepared that suicide may occur in connection with an admission, they may react with shock and be affected emotionally. The fact that professionals are affected when a patient they have followed up commits suicide may possibly enhance their capacity to confirm bereaved relatives. The professional’s ability to recognize their own emotions is a condition for building bridges so that family members perceive that they are seen, understood and less lonely. Encounters characterized by equal status can free up energy that makes it possible to accept confirmation.^26,34^ Encountering family members as equals by giving something of themselves creates a basis for dialogue.

Suffering requires time and space.^23,28^ One of the quotations describes family members verbally attacking an employee, whom the participant eventually stops in order to protect the colleague. Stopping the bereaved from expressing emotions may increase suffering because absence of confirmation of suffering places the person’s credibility and dignity in question.^22,23^ But intervening in locked situations may also be seen as protecting the person’s dignity. Demanding conversations like this one challenge the ability of professionals to distinguish between their own needs and those of the family members, according Dransart et al.^4^ Even when a patient’s suicide ideation has been a topic that has concerned both family members and professionals, the suicide may come as a shock to the professionals as well. Like family members, they can also be affected by feelings of guilt,^4^ and errors and deficiencies can occur in risk assessment and treatment. To be able to confirm those who were bereaved by suicide in this phase requires that health professionals’ can regulate their own feelings and reactions.

Since MHCPs are one of the groups that encounters family members during the critical phase following a suicide, usually the first 24 h or days, they can play a crucial role. In line with Norton,^5^ the first responders have the opportunity to frame and influence a response without guilt and shame. The theme *Creating an encounter through dialogue* is about how the participants in this study tried to achieve dialogues in cases where the situation was initially locked. This act is understood as an expression of the participants’ efforts to listen, confirm and inform in order to create an encounter that can alleviate and relieve the suffering of the family members. The participants seem to use the procedure found in national guidelines^6^ as a starting point for dialogue relating to the suicide. The guidelines recommend that relatives receive correct and prompt information, time for questions, offers of conversations with the treatment personnel and other staff members who have been in contact with the patient, etc. As the statements illustrate, participants also provide time and space for the family members’ intense feelings about what has happened. They share their own perception of what happened, and in addition listen to the bereaved. The participants appear to be led more by the moral responsibility that grows through witnessing the suffering of the bereaved than by policies, procedures and time resources. They are regarded as working in accordance with Eriksson’s^23,29^ mantra: ‘I was there, I saw, I witnessed, and I became responsible’. Caregiving appears to be wholehearted. Being left to oneself reinforces suffering,^22,23^ particularly in an early phase after the suicide. The bereaved appreciate fellowship and conversations with someone who understands the incident.^11,17,18^ Dialogue can be compromised when the MHCPs are challenged to be able to look past the aggressive expressions of family members. Lindström^26^ points out that our tendency to focus on the negative aspects of aggression may be due to aggression often being accompanied by feelings of anxiety and guilt. The contrary of aggression is isolation and loss of contact. Many family members are tired of long-term concern and fear and are relieved that the patient has finally been admitted.^31^ Perhaps that is why the suicide may come to them like ‘a bolt from the blue’ and trigger anger and aggression. Clarke and Ebert^19^ are of the opinion that many people perceive suicide as a curable disease, making communication between relatives and professionals particularly challenging. In light of this explanation, the aggressive reactions of the family members take on an expanded meaning: The suicide is seen as a result of inadequate treatment, which triggers a need for retaliation. Listening involves an exploration of underlying meaning in what is expressed. Shame and thoughts of responsibility and guilt for not seeing possible signs that could have averted the suicide are reactions that many bereaved by suicide need professional help to process.^14,16^

It takes time to accept a different reality,^16^ as this study confirms. The grieving process involves work to re-establish identity and relations^9^ and to reconcile oneself with the fact that a suicide has taken place. Under the theme *Providing consolation and reconciliation,* experiences are documented and indicate that family members may need consolation and various forms of acute help but also help over a long period of time. Consolation relieves and alleviates suffering, as it awakens confidence and hope^22,23,27,35^ Hope as ‘an inner flame’ gives strength to endure suffering, and hoping is a consoling experience.^36^ In order to achieve a consoling dialogue, both the consoler and the sufferer must be ready for consolation.^35^ Experiences in this study show that this can be demanding to achieve right after the suicide.

The participants in this study go far beyond the framework and guidelines for making oneself available to family members following suicide. According to Eriksson, ethics always happen here and now.^29^ Being present seems to awake the professionals’ responsibility for what they saw and understood. While more long-term follow-up after suicide in Norway has been assigned to the municipal health service,^3^ this study argues for follow-up by those who knew the patient, when this is the wish of the family members. However, there are cases, as the statements show, where the bereaved ones’ need other, neutral interlocutors, where the task is to ensure rapid follow-up with the possibility of consoling dialogue. The fact that someone is listening provides the strength to share and explore thoughts and feelings.^28^ An encounter with professionals who can tell what they have seen in the deceased person and how they have been affected by him or her, not only as a suicidal patient, can be a source of consolation for the bereaved. When the participants tell family members what they admired in the deceased, they convey themselves as caring human beings. The encounter as a caring act requires courageous and secure healthcare professionals who dare to give of themselves.^28,34^ The general practitioner who has followed the patient before the suicide can be an important support for the grieving family. The danger is that physical issues as a result of grieving may overshadow family members' struggle with thoughts and feelings and perhaps also suicidal ideation.^19^ Encountering other bereaved who have moved forward in their own care process is perceived as an important element of support. Shared experience makes it easier for family members to share their own narrative and feel that they are understood.^11,16–18^ Such conversations can be experienced as comforting and also provide hope for the future.

Consolation includes help and guidance.^27^ Participants speak about family members who struggle with questions about the suicide for a long time after the suicide, and about a high threshold to ask for help. It seems very important that offers are made repeatedly and that professionals initiate contact. Bereaved by suicide, not least children and young people, need ‘tailored’ care because their need for care and support changes in their process.^15,16^ According to Bergbom et al.,^24^ reconciliation is about creating and transforming an entity that includes the evil in a new meaningful wholeness.

As we see it, understanding a matter such as suicide can contribute to consolation and also to reconciliation. The process of reconciliation can be understood in light of Gadamer’s^21^ hermeneutics: The bereaved’s understanding of the suicide is created through the process which explores and assembles fragments of their own and others’ understanding of the person’s life, suffering, and his or her relationship with the outside world.

## Strengths and limitations

The six participants who reported experiences about encounters with family members following suicide represented both men and woman and belonged to five different units. Data in this study were produced in an atmosphere characterized by openness and trust, where the moral responsibility seemed to be the guiding star for the participants. Based on content and depth, data were considered rich enough to answer the research question. Participants were recruited from only two hospitals. Experiences from several hospitals could possibly have added more nuances to the themes. Findings in this study are considered valid and reliable, based on the chosen methodology and theoretical perspectives. In line with Kvale and Brinkmann,^32^ to validate is to reflect and control through all stages in the research process.

The researchers’ own preunderstanding may represent an obstacle, while at the same time, preunderstanding represents a positive premise for a different understanding.^21^ Although we argue that the first author’s preunderstanding through working with suicidal patients in a context of mental health prevention has a positive impact on the conduct of the study, it is crucial to be critically aware of one’s preunderstanding throughout the research process.

The project was led by the first author, who also conducted the interviews. The co-authors participated in the project from planning to publication of the results. The project has had an external resource group consisting of participants representing patient and user experiences, family member’s experiences, clinical experiences and research. The group consented to follow the project and have offered input, via regular meetings with the researchers.

## Conclusion

The participants in this study aimed to go far beyond the first phase after the suicide in encountering family members following suicide. They appear to be led by their moral responsibilities. Findings illustrate a way by which MHCPs can care for those bereaved in the context of psychiatric wards. The professionals seek to confirm suffering, to create dialogue even when it is initially locked. They seek to comfort and to support so as to initiate a movement through suffering towards reconciliation and thereby health. It is both an ethical and a professional challenge to provide care for family members following suicide when suffering is expressed through aggression and accusations directed at treatment and staff.

Many family members who are bereaved by suicide will need help from both professionals and peers to create new hope and new meaning in everyday life. Caring may prevent suffering as well as suicide among those who were bereaved by suicide. Caring for family members following suicide must be given priority in mental health services. This study brings new knowledge to a demanding topic that is sparsely explored. Even though, this field needs more research for a better understanding of the family members’ different reactions and to encounter the bereaved when a patient commits suicide while hospitalized.
